# Draft Genome Sequence of Helicobacter cinaedi, Compiled by Direct Whole-Genome Sequencing of a Blood Culture-Positive Isolate in Hong Kong

**DOI:** 10.1128/MRA.01263-18

**Published:** 2018-11-08

**Authors:** Wai-Sing Chan, Chun-Hang Au, Henry Chi-Ming Leung, Dona N. Ho, Tsun-Leung Chan, Tak-Wah Lam, Edmond Shiu-Kwan Ma, Bone Siu-Fai Tang

**Affiliations:** a Department of Pathology, Hong Kong Sanatorium & Hospital, Hong Kong, China; b Department of Computer Science, The University of Hong Kong, Hong Kong, China; c L3 Bioinformatics Limited, Hong Kong, China; Loyola University Chicago

## Abstract

Isolation of Helicobacter cinaedi from a positive blood culture requires prolonged and stringent subculture conditions. Direct whole-genome sequencing (WGS) of a positive blood culture may provide timely treatment-associated genetic information. Here, we report a draft genome sequence of H. cinaedi compiled by direct WGS, which was 1,995,911 bp in length with 39.1% GC content.

## Abstract (precis)

Isolation of Helicobacter cinaedi from a positive blood culture requires prolonged and stringent subculture conditions. Direct whole-genome sequencing (WGS) of a positive blood culture may provide timely treatment-associated genetic information.

## ANNOUNCEMENT

We report here the draft genome sequence of a Helicobacter cinaedi isolate from a patient with bacteremia. The patient was a 44-year-old gentleman who suffered from glioblastoma multiforme of the brain and diffuse large B-cell lymphoma of cervical lymph nodes. He initially received radiotherapy to the brain and cervical lymph nodes followed by chemotherapy consisting of rituximab and cyclophosphamide, doxorubicin hydrochloride, vincristine sulfate, and prednisolone (R-CHOP). A blood culture was performed on 17 September 2016 and became positive after 4 days. A follow-up subculture revealed pure growth of H. cinaedi with MICs of 1 μg/ml and 0.02 μg/ml for amoxicillin-clavulanate and meropenem, respectively. Identification and antibiotic susceptibility reports were released 12 days after subculture. This time lapse reflects challenges of characterizing fastidious bacteria from positive blood cultures, and it formed the basis of this study, in which we speculated that this bottleneck might be alleviated by direct whole-genome sequencing (WGS). Furthermore, characterization of H. cinaedi from a genomic perspective may contribute to a better understanding of this emerging human pathogen.

Bacterial DNA was directly extracted from 1 ml of positive blood culture using a NucliSENS easyMAG system and sequenced with Nanopore MinION and Illumina MiSeq technology. For MinION analysis, DNA was amplified at 30°C for 2 h using a Repli-g midikit (Qiagen) to increase input for 1-dimensional (1D) library preparation. Real-time sequencing was performed on a FLO-MIN106 R9 flow cell (Oxford Nanopore Technologies). For MiSeq analysis, 0.2 ng/µl DNA was subjected to Nextera XT library preparation (Illumina) and sequenced in a 2 × 300-bp paired-end run using a MiSeq v3 reagent kit (Illumina).

Totals of 102,461 and 5,890,308 reads were generated by MinION and MiSeq runs, respectively. Sequencing reads were quality filtered using Trimmomatic (Galaxy version 0.36.0) (1). Human reads were filtered by mapping to genome assembly hg19 using the Burrows-Wheeler Aligner MEM algorithm (UGENE version 1.29.0) (2). Hybrid assembly of MinION and MiSeq reads was generated by Unicycler with default parameters (Galaxy version 0.4.4.0) (3). Genome quality was assessed using QUAST (Galaxy version 4.6.3) (4). The assembly comprised 24 contigs with a total length of 1,987,098 bp and an *N*_50_ value of 187,636 bp. Nineteen contigs were longer than 500 bp, and the largest was 329,137 bp. MeDuSa was used for reference-guided scaffolding (GenBank accession numbers AP017374, NC_017761, and NC_020555) (5). Genome annotation was facilitated by the NCBI Prokaryotic Genome Annotation Pipeline (6). The draft genome contained 1,995,911 bp and 39.1% GC content, and it was predicted to possess 1,858 protein-coding genes, 39 tRNAs, and 2 each 5S, 16S, and 23S rRNA genes, with 78.0× genome coverage. It harbored virulence-associated *cdt* and *ahpC* genes and two CRISPR arrays.

By MinION analysis, we identified H. cinaedi and a 23S rRNA gene mutation associated with clarithromycin resistance (2018A→G, 13× coverage comprising 85% guanine) (7, 8) after the first 15 min and 2 h of the sequencing run, respectively. This mutation was also present in the MiSeq-derived sequence. With sufficient sequencing depth and knowledge of resistance mechanisms, direct WGS may complement conventional methods for quick characterization of fastidious etiologic agents of bacteremia.

### Data availability.

This draft genome sequence of Helicobacter cinaedi has been deposited in NCBI GenBank under accession numbers NZ_CP029337 and CP029337. The Illumina data have been deposited in the NCBI Sequence Read Archive under accession number PRJNA436201.
